# Removal of Fragment of Steel from Vitreous by Electro-Magnet

**Published:** 1891-06

**Authors:** 


### REMOVAL OF FRAGMENT OF STEEL FROM VITREOUS BY ELECTRO-MAGET.

Dr. Charles Barck stated, the case he was about to report was one of successful extraction of a piece of steel from the vitreous humor by means of an electro-magnet. Its interest resides chiefly in the unusually good vision that has been finally obtained. The patient is forty years of age; his left eye received a severe injury about fifteen years ago, in consequence of which his vision with it is very poor. On November 13th of last year, his right eye was pierced with a piece of steel. He was under the care of an oculist in this city for eight days; the speaker saw him first on November 21. There was then a small horizontal scar at the inner upper margin of the cornea about one line long; corresponding to this, there was a small perforation of the iris, and at the point of perforation adhesion to the lens capsule; the pupil was dilated in consequence of the instillation of atropine. Nearly the whole lens was opaque from traumatic cataract, a streak was visible extending from the anterior to the posterior lens capsule, from upward somewhat downward, clearly indicating the direction the foreign body had traveled, besides in the vitreous there was a small opacity with a somewhat metallic gray, yellowish reflex. The opacity and the reflex in the vitreous could not be seen very distinctly on account of the existing cataract, yet the conclusion was authorized that the foreign body had passed through the cornea, iris and lens and was located in the vitreous. The same diagnosis was arrived at by Dr. Hunicke, of this city. We therefore concluded, if possible, to remove the foreign body from the vitreous. The operation was performed the next day, November 22. The corneal section was made downward, an iridectomy done, the debris of the lens opened, and the electro-magnet introduced. The foreign body was located deeper than we had at first supposed, and only after the introduction of the magnet several times was the foreign body secured and removed. It was about one line long, and a half line wide. The healing process was regular and uninterrupted; opaque masses of the lens which were left gradually underwent partial absorption, but much was still retained. On March 1 this was operated on by the needle operation and a tolerably round pupil was obtained. His vision is $\frac{20}{40}$ with convex glass or twelve dioptrics, and with a convex lens of fifteen dioptrics, he reads Snellen No. 5. The vitreous is completely clear and transparent and the fundus can be recognized distinctly.—*St. Louis Medical and Surgical Journal, May, 1891.*

